# Effect of integrated nursing on serum inflammatory factors and oxygenation indices in children with severe respiratory failure: A prospective quasi-experimental controlled study

**DOI:** 10.1097/MD.0000000000048866

**Published:** 2026-05-29

**Authors:** Huifen Zhang, Yanhe Ma, Lihong Hu, Yuxiang Wang, Na Zhang, Meixian Xu

**Affiliations:** a Department of Pediatric Intensive Care Unit, Hebei Children’s Hospital, Shijiazhuang, Hebei, China; b Department of Critical Care Medicine, Hebei Provincial Clinical Research Center for Child Health and Disease, Shijiazhuang, Hebei, China.

**Keywords:** inflammation, intensive care nursing, mechanical ventilation, oxygenation, pediatric respiratory failure

## Abstract

This study aimed to evaluate the effects of an integrated nursing model on systemic inflammation, oxygenation, and clinical outcomes in children with severe respiratory failure. Children with severe respiratory failure requiring invasive mechanical ventilation for ≥72 hours were consecutively enrolled. Participants were allocated according to admission period into a routine nursing group (2023) and an integrated nursing group (2024–2025). The integrated nursing model involved multidisciplinary assessment, coordinated care planning, and continuous nursing evaluation. Primary outcomes included changes in inflammatory markers (interleukin-6, tumor necrosis factor alpha, and C-reactive protein), while secondary outcomes included oxygenation indices (PaO_2_/FiO_2_), duration of mechanical ventilation, pediatric intensive care unit length of stay, and complications. Data were analyzed using independent-sample tests, chi-square tests, and linear mixed-effects models. Compared with routine nursing, integrated nursing was associated with greater reductions in interleukin-6, tumor necrosis factor alpha, and C-reactive protein over time and faster improvements in oxygenation indices. The integrated care group also had shorter mechanical ventilation duration and pediatric intensive care unit length of stay, and a lower incidence of ventilator-associated pneumonia. Integrated nursing may improve inflammatory control, oxygenation, and clinical outcomes in critically ill children with severe respiratory failure. Further multicenter randomized studies are warranted.

## 1. Introduction

Pediatric severe respiratory failure represents a critical condition often seen in pediatric intensive care units (PICUs).^[1]^ The underlying mechanisms primarily involve severe oxygen-transfer problems caused by lung inflammation and damage.^[2]^ Even with ongoing improvements in life-support technologies like breathing machines, children facing this condition still experience high death rates and possible long-term health issues.^[3]^

Traditional critical care nursing approaches frequently focus on discrete, task-focused actions, which often suffer from poor teamwork across different medical areas.^[4]^ Additionally, nursing actions may not fully align with the child’s changing situation and personal needs. This could mean worse control of inflammatory responses, more injuries from breathing machines, and other problems, affecting the overall recovery chances.^[5]^

In recent times, the rise of “whole-person medicine” has pushed changes in nursing models, focusing on the patient as the center.^[6]^ This model integrates knowledge from many fields, continuous monitoring, and personalized care plans to provide comprehensive, well-coordinated care.^[7,8]^ In this study, integrated nursing refers to a structured, multidisciplinary nursing approach led by specialized nurses in collaboration with physicians, respiratory therapists, pharmacists, and nutrition specialists. This model emphasizes comprehensive patient assessment, coordinated decision-making, proactive risk management, and individualized care planning to optimize respiratory support, infection prevention, and nutritional management in critically ill children.

For adults, this whole nursing model has shown value in improving outcomes and boosting recovery in long-term illnesses and in elderly care.^[9]^ However, how it works and its effects in the urgent, complex setting of pediatric breathing failure remain largely unknown. Therefore, this study aimed to evaluate the effects of an integrated nursing model on systemic inflammatory responses, oxygenation indices, and clinical outcomes in children with severe respiratory failure in the PICU. It will focus on changes in key blood markers of inflammation, such as interleukin-6 (IL-6), tumor necrosis factor alpha (TNF-α), C-reactive protein (CRP), and oxygen levels. It will also assess links to real-world evidence, such as the duration of breathing support and associated complications, providing valuable insights to improve nursing care for this serious pediatric condition. Therefore, the research question of this study was whether an integrated nursing model could improve inflammatory control, oxygenation status, and clinical outcomes in children with severe respiratory failure compared with routine nursing care. We hypothesized that the integrated nursing intervention would lead to greater reductions in inflammatory markers and improved clinical outcomes.

## 2. Materials and methods

### 2.1. Study subjects

#### 2.1.1. Inclusion and exclusion criteria

This study was conducted prospectively from January 2023 to January 2025, and data for both groups were collected prospectively during hospitalization. Severe respiratory failure was defined according to the Guidelines for the Diagnosis and Treatment of Pediatric Acute Respiratory Distress Syndrome (2023 Edition): acute-onset respiratory distress without primary cardiac failure, new or worsening bilateral infiltrates on chest imaging, and PaO_2_/FiO_2_ ≤ 200 mm Hg under invasive mechanical ventilation. Only children expected to require invasive mechanical ventilation for ≥ 72 hours were included. Written informed consent was obtained from each participant’s legal guardian before enrollment.

Children were eligible for inclusion if they met the following criteria: diagnosed with severe respiratory failure according to the Guidelines for the Diagnosis and Treatment of Pediatric Acute Respiratory Distress Syndrome (2023 edition); required invasive mechanical ventilation and were expected to receive ventilatory support for at least 72 hours; and admitted to the PICU during the study period.

To minimize the impact of confounding factors, the following exclusion criteria were set up: children over 14 years old or <28 days of age (neonates); having congenital heart diseases, serious neurodevelopmental disorders, or life-limiting illnesses; hospital-acquired infections or colonization with multidrug-resistant organisms prior to admission; and extreme hemodynamic instability requiring continuous high-dose vasoactive agents. After applying these exclusion criteria, participants were allocated according to admission period. Written informed consent was obtained from the legal guardians of all eligible participants before enrollment. This study was approved by the Ethics Committee of Hebei Children’s Hospital (Approval No. 202407-71).

#### 2.1.2. Grouping design

Children admitted from January to December 2023 received routine nursing care and served as the control group. After the integrated nursing model was implemented as standard practice, children admitted from January 2024 to January 2025 received integrated nursing in addition to standard treatment and served as the integrated care group. During both admission periods, the PICU followed the same institutional protocols for ventilatory management, sedation/analgesia, antimicrobial stewardship, and nutrition support. No major guideline updates or staffing model changes were implemented between the 2 periods. Laboratory assays were performed using the same platforms and internal quality-control procedures throughout the study.

#### 2.1.3. Sample size calculation and grouping method

The primary focus of this study was changes in serum IL-6 levels after patients received the combined nursing care approach. Based on a pilot assessment in our unit and a review of published literature, the average postintervention IL-6 level in the routine care group was estimated to be approximately (25.0 ± 8.0) pg/mL, and we anticipated a reduction to (18.0 ± 6.5) pg/mL in the integrated nursing group.^[10]^ The study planning involved setting the power (1 − -β) to 0.80, and we used a 2-sided α level of 0.05 for the statistical analysis. Using the PASS 15.0 tool (NCSS, LLC, Kaysville) to determine the sample size for comparing 2 independent groups, we calculated that we needed at least 60 cases per group. To account for potential missing data, we inflated the sample size by 20%, resulting in a planned total of 150 participants (75 per group). Participants were allocated to groups based on admission period as described above. Eligible children were consecutively enrolled during the study period and were allocated to groups according to the prespecified admission periods.

### 2.2. Intervention protocol

#### 2.2.1. Integrated nursing implementation process

The comprehensive nursing approach used for the research participants represents a coordinated, multi-stage process involving different healthcare professionals working together. This integrated nursing model was initiated within 24 hours of PICU admission and delivered as a structured multidisciplinary care pathway. The primary nurse carries out an initial “comprehensive nursing evaluation” using a specially designed tool. This evaluation examines the child’s body functions, such as breathing and blood circulation; nutritional status; skin condition; emotional reactions; behavior; family support networks; and possible care-related issues.

Based on this evaluation, a temporary care team is formed, including the doctor in charge, specialized nurses, breathing specialists, pharmacy experts, and nutrition staff. This group holds case discussions twice per week, applying both medical research findings and practical know-how to create and adjust personalized care plans for each patient. These plans cover not just breathing support strategies, pain management approaches, and food intake support, but also place strong emphasis on teaching families and providing emotional support. Furthermore, a visual tool helps map out complex care steps, ensuring everyone on the team and family members clearly understand the care goals and methods. The primary nurse serves as the main coordinator, responsible for organizing the plan, implementing it, and documenting all activities.

#### 2.2.2. Control group routine nursing protocol

Children in the control group receive standard care in the PICU. This care follows the department’s guidelines, which focus on the disease and physician orders. The attending nurse carries out tasks as directed by the physician. These tasks include monitoring of vital signs on a routine basis, setting parameters for providing mechanical support for breathing and recording these values, completing administration of medication on schedule, performing basic care tasks that include repositioning, tapping on the back, and care of the oral area, and providing removal of secretions as needed. The implementation of care measures is based on the individual nurse’s experience and the department’s standard practices. It does not include a systematic approach that provides proactive, multi-dimensional assessment tailored to the individual patient. Consultations with specialists are requested through the traditional, on-demand model. This means that relevant specialists are invited for consultation only when clear complications or challenges in treatment arise. Communication and collaboration between disciplines are relatively passive and fragmented. Communication with the family focuses on providing information about the condition and general health education. It lacks a structured plan for continuous family involvement and support.

#### 2.2.3. Intervention duration and assessment points

We defined the intervention period as the interval from enrollment – when the child began the assigned nursing protocol – to successful discontinuation of invasive mechanical ventilation and transfer out of the PICU. To systematically evaluate intervention effects, we specified 4 assessment time points. Research assistants collected baseline data within 24 hours of enrollment (T0). They assessed early inflammatory responses and oxygenation changes on day 3 after initiating the intervention (T1, mid-intervention). They evaluated sustained intervention effects on day 7 (T2, late-intervention). They completed outcome assessments within 24 hours before successful extubation and transfer out of the PICU (T3, endpoint). The same data collection procedures and assessment time points were applied to both the control group and the integrated nursing group to ensure consistency across groups.

At each time point, the research assistants obtained venous blood samples to measure serum inflammatory markers (IL-6, TNF-α, and CRP). They recorded arterial blood gas results and calculated oxygenation indices (e.g., PaO_2_/FiO_2_). Throughout the intervention period, the study team also tracked mechanical ventilation duration, PICU length of stay, and the incidence of ventilator-associated pneumonia (VAP) and other complications. We trained all research assistants using a standardized program and kept them independent from the nursing interventions to reduce measurement bias and maintain data objectivity.

### 2.3. Study indicators

#### 2.3.1. Primary indicator: serum inflammatory markers

ELISA measured serum inflammatory markers IL-6 and TNF-α, and CRP was quantified by immunoturbidimetric assay. Blood samples were collected strictly at the T0 to T3 time points (morning fasting) and processed by centrifugation and storage at −80°C. The samples were then analyzed by blinded laboratory personnel. The results were reported in pg/mL for IL-6 and TNF-α, and mg/L for CRP, to assess the level of systemic inflammatory response.

#### 2.3.2. Secondary indicators: oxygenation indices

1. PaO_2_/FiO_2_: PaO_2_ was measured using an arterial blood gas analyzer, and the FiO_2_ was derived from the ventilator records, allowing for the calculation of the PaO_2_/FiO_2_ ratio.
2. SpO_2_: Continuous monitoring by the bedside monitor provided daily average SpO_2_ values.

All measurements were taken at a fixed time each day, during a stable state for the child, to ensure data comparability and to reflect lung oxygenation function objectively.

#### 2.3.3. Clinical outcome indicators

1. Mechanical ventilation duration and PICU length of stay: The research team recorded mechanical ventilation duration as the total hours from endotracheal intubation to successful extubation. They calculated PICU length of stay as the total number of days from PICU admission to discharge.
2. Complications: The clinical team diagnosed VAP using the 2016 Infectious Diseases Society of America/American Thoracic Society criteria adapted for pediatric patients. They confirmed pneumothorax by bedside chest X-ray or ultrasound. They defined catheter-associated bloodstream infection according to the US Centers for Disease Control and Prevention criteria. Two independent physicians who did not participate in the study reviewed all suspected complications and reached consensus on the final diagnoses. The study team reported complication outcomes as counts and percentages (%).

#### 2.3.4. Safety indicators

Serious events in the study include those that produce death, conditions that threaten life, hospitalization, or hospitalization that continues for extended periods, or disability that persists or shows severe loss of function. Events that do not reach this level are also recorded in the study. These include injuries to the skin from pressure that relate to nursing procedures, following the categories provided by the National Pressure Injury Advisory Panel in its guidance documents. The study also recorded interruptions to treatment that occur without planning, such as accidental removal of breathing tubes. Clinical deterioration potentially related to the intervention was also recorded. This includes new cases of severe instability in blood flow patterns, suppression of breathing function, and similar conditions. Review of all events that occur in the study follows a regular schedule. The research group conducted scheduled audits of medical records to identify and adjudicate adverse events. The assessment of the association between events and the intervention in the study involves a joint evaluation. The investigator who leads the study and a physician conduct this assessment. This physician does not participate in the nursing interventions examined in the study.

### 2.4. Statistical analysis

All study data were recorded using standardized electronic case report forms. Two trained research nurses who were independent of the intervention team were responsible for data collection. The database was established using Microsoft Excel and subsequently analyzed using SPSS version 26.0 (IBM Corp., Armonk). Data accuracy was ensured through double-entry verification and periodic review by the research team.

We summarized normally distributed continuous variables as mean ± standard deviation (*x*ˉ ± *s*) and compared groups using the independent-samples *t* test. For non-normally distributed variables, we reported the median and interquartile range and used the Mann–Whitney *U* test for between-group comparisons. We presented categorical variables as counts and percentages (n [%]) and compared proportions using the chi-square test or Fisher exact test, as appropriate. The normality of continuous variables was assessed using the Shapiro–Wilk test. Models were additionally adjusted for age, sex, primary diagnosis category, pediatric critical illness , and baseline PaO_2_/FiO_2_.

To evaluate changes over time, we used linear mixed-effects models with fixed effects for group, time, and group-by-time interaction and a random intercept for participants. For time-point-specific between-group comparisons, *P* values were adjusted using the Bonferroni method.

## 3. Results

### 3.1. Comparison of baseline characteristics between the 2 groups

A total of 150 children were included in this study: 75 in the control group and 75 in the study group, with no dropouts. Baseline characteristics were similar between the 2 groups (Table 1).

**Table 1:** Comparison of baseline characteristics between the 2 groups.

Characteristic	Control group (n = 75)	Integrated care group (n = 75)	*t*/χ^2^/*Z* value
Age (months), M (IQR)	24.0 (12.0, 48.0)	26.0 (13.5, 52.5)	*Z* = −0.521
Male, n (%)	42 (56.0)	45 (60.0)	χ^2^ = 0.267
Weight (kg), *x*ˉ ± *s*	12.5 ± 4.2	13.1 ± 4.8	*t* = −0.826
Primary diagnosis, n (%)			χ^2^ = 0.894
Severe pneumonia	38 (50.7)	35 (46.7)	
ARDS	22 (29.3)	25 (33.3)	
Sepsis	15 (20.0)	15 (20.0)	
PCIS, *x*ˉ ± *s*	72.3 ± 8.5	73.1 ± 7.9	*t* = −0.589
Baseline PaO_2_/FiO_2_ (mm Hg), *x*ˉ ± *s*	152.6 ± 28.4	148.9 ± 31.7	*t* = 0.731

ARDS = acute respiratory distress syndrome, M (IQR) = median (interquartile range), PCIS = pediatric critical illness score, *x*ˉ ± *s* = mean ± standard deviation.

### 3.2. Trends in the change of inflammatory marker levels

**Figure 1.**
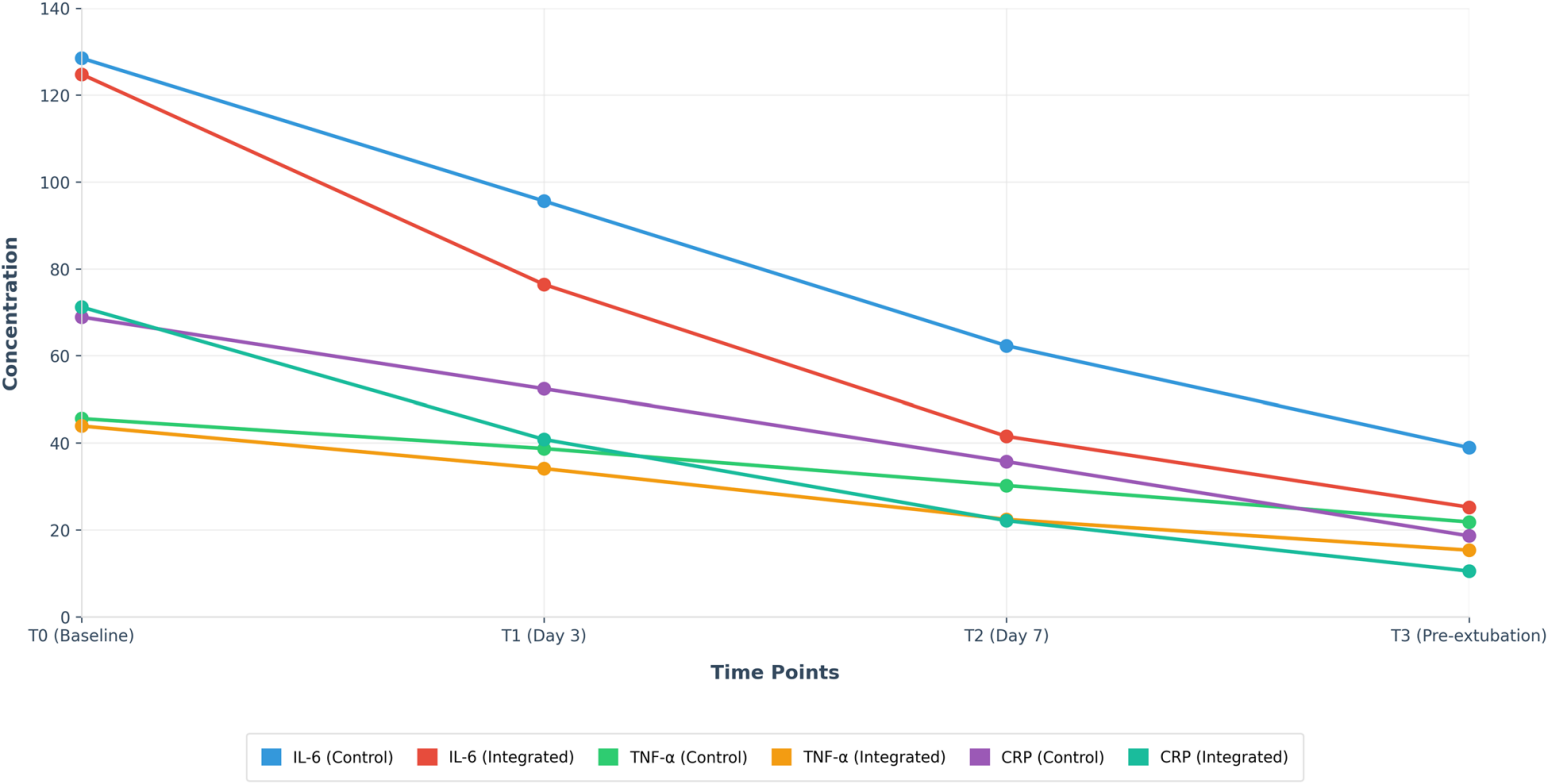
Serum inflammatory factor levels over time. CRP = C-reactive protein, IL-6 = interleukin-6, TNF-α = tumor necrosis factor alpha.

At baseline (T0), IL-6, TNF-α, and CRP levels did not differ significantly between groups (all *P* > .05). Over time, inflammatory markers decreased in both groups, with a significantly greater reduction in the integrated nursing group. As the care period continued, however, the group receiving the integrated nursing approach demonstrated a faster and more pronounced reduction in all 3 substances, as illustrated in Figure 1. Linear mixed-effects models showed significant group-by-time interactions for IL-6, TNF-α, and CRP (all *P* < .001), indicating a greater reduction over time in the integrated nursing group. Further examination revealed that, starting on the 3rd day of the intervention period (T1), IL-6 and CRP levels in the integrated nursing group were significantly lower than in the control group (*P* < .05). Then, by the 7h day (T2) and just before tube removal (T3), all 3 markers – IL-6, TNF-α, and CRP – were significantly lower in the integrated nursing group than in the control group (*P* < .01)(see Table 2).

**Table 2:** Comparison of serum inflammatory factor levels at different time points.

Inflammatory factor	Time point	Control group (n = 75)	Integrated care group (n = 75)	*t* value	*P* value
IL-6 (pg/mL)	T0 (baseline)	128.5 ± 42.3	124.8 ± 38.6	0.562	.575
	T1 (day 3)	95.6 ± 35.7	76.4 ± 28.9	3.672	<.001
	T2 (day 7)	62.3 ± 22.1	41.5 ± 18.7	6.245	<.001
	T3 (preextubation)	38.9 ± 16.4	25.2 ± 12.6	5.789	<.001
TNF-α (pg/mL)	T0 (baseline)	45.6 ± 15.2	43.9 ± 14.5	0.703	.483
	T1 (day 3)	38.7 ± 12.8	34.1 ± 11.3	2.343	.021
	T2 (day 7)	30.2 ± 10.5	22.4 ± 8.6	4.912	<.001
	T3 (preextubation)	21.8 ± 9.1	15.3 ± 6.8	4.876	<.001
CRP (mg/L)	T0 (baseline)	68.9 ± 24.5	71.2 ± 26.3	−0.557	.578
	T1 (day 3)	52.4 ± 20.1	40.8 ± 17.6	3.683	<.001
	T2 (day 7)	35.7 ± 15.3	22.1 ± 11.4	6.194	<.001
	T3 (preextubation)	18.6 ± 9.8	10.5 ± 6.2	6.025	<.001

Data are presented as mean ± standard deviation.

CRP = C-reactive protein , IL-6 = interleukin-6, TNF-α = tumor necrosis factor-alpha.

### 3.3. Improvement of oxygenation indices

**Figure 2.**
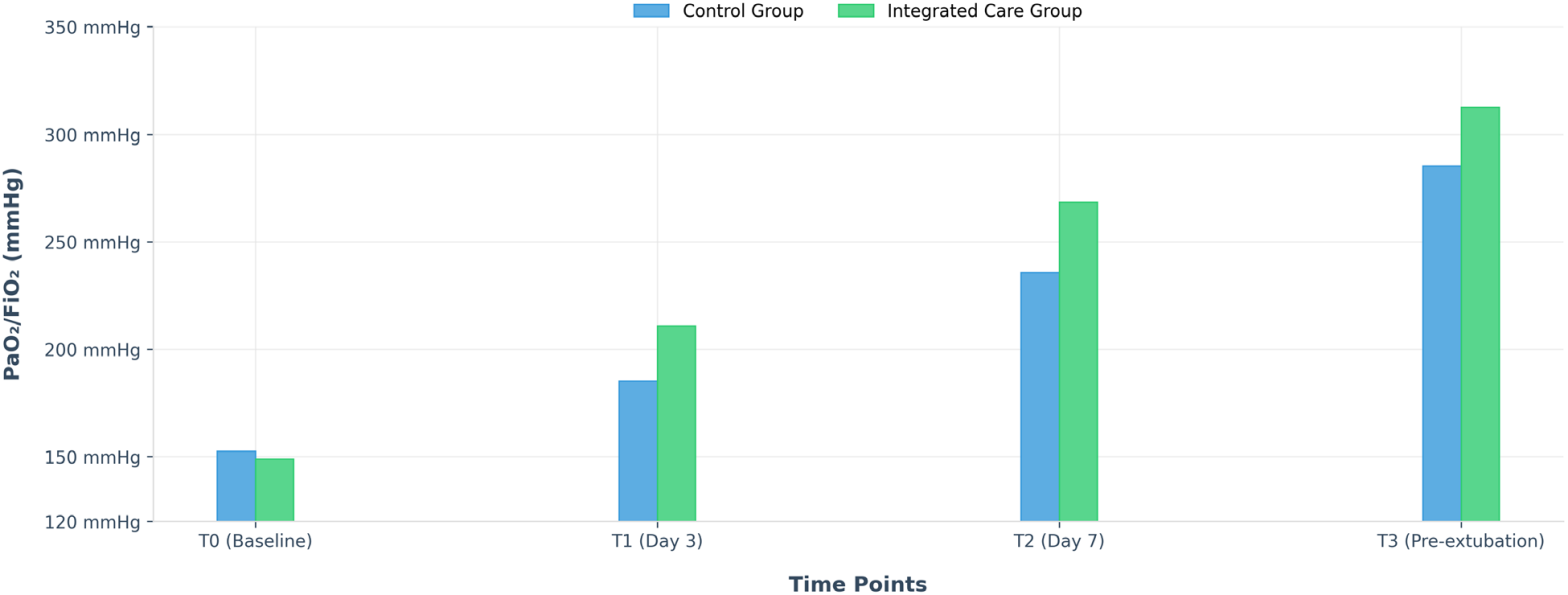
Oxygenation index (PaO_2_/FiO_2_) over time.

At the beginning of the study, both sets of patients showed PaO_2_/FiO_2_ levels indicative of severe breathing problems, with no significant differences between them at that time. Over the course of treatment, as shown in Table 3 and Figure 2, both groups showed higher PaO_2_/FiO_2_ ratios over time, indicating improvement. However, the group that received the combined care approach improved much faster and to a greater extent than the other group. When we looked at how the numbers changed over time and across groups, a statistical method identified a significant association between the time factor and the patients’ group. From day 3 (T1) onward, the integrated care group showed significantly higher PaO_2_/FiO_2_ values than the control group, and the between-group difference increased over time. The between-group difference increased over time. At the later checkpoints, T2 and T3, the difference became very large and highly significant. Furthermore, the combined nursing group also had higher average daily oxygen saturation levels throughout the period; that is, their SpO_2_ readings were consistently higher than those of the control group.

**Table 3:** Comparison of oxygenation index (PaO_2_/FiO_2_) at different time points.

Time point	Control group (n = 75)	Integrated care group (n = 75)	*t* value	*P* value
T0 (baseline)	152.6 ± 28.4	148.9 ± 31.7	0.731	.466
T1 (day 3)	185.3 ± 35.2	210.8 ± 32.6	−4.627	<.001
T2 (day 7)	235.7 ± 41.8	268.5 ± 39.1	−4.879	<.001
T3 (preextubation)	285.4 ± 45.3	312.6 ± 42.7	−3.691	<.001

Data are presented as mean ± standard deviation (mm Hg).

### 3.4. Comparison of clinical outcome indicators

**Figure 3.**
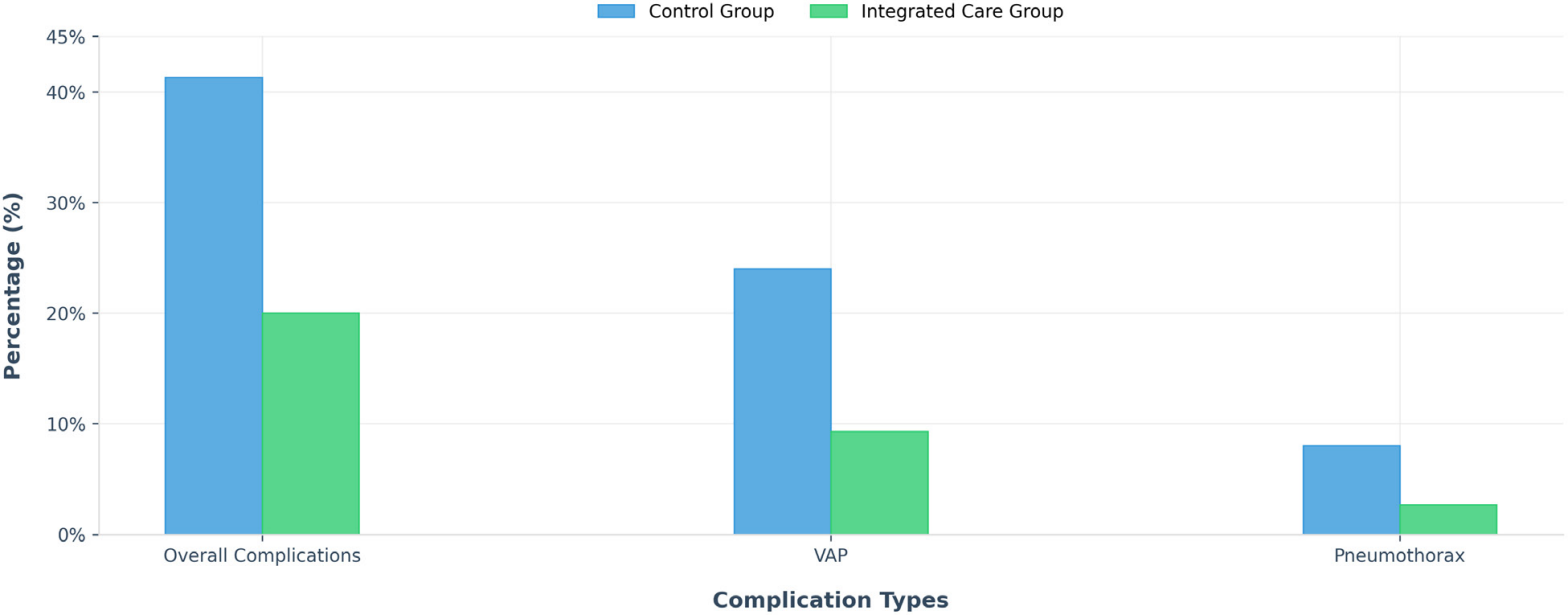
Clinical outcomes comparison. VAP = ventilator-associated pneumonia.

The integrated nursing group had a significantly shorter duration of mechanical ventilation and PICU stay. The overall complication rate was also lower, particularly the incidence of ventilator-associated pneumonia. As shown in Table 4 and Figure 3, the overall complication rate was much lower in the integrated nursing group (20.0%) than in the control group (41.3%). For instance, pneumonia linked to the breathing machine occurred significantly less often in the integrated group (9.3% compared to 24.0%). While the occurrence of air leaks around the lung also seemed lower (2.7% vs 8.0%), this difference was not significant enough to rule out a chance. Importantly, bloodstream infections associated with tubes were not observed in either patient group.

**Table 4:** Comparison of clinical outcomes between the 2 groups.

Outcome measure	Control group (n = 75)	Integrated care group (n = 75)	Statistical value	*P* value
Duration of mechanical ventilation (hours), M (IQR)	168.0 (120.0, 216.0)	120.0 (96.0, 168.0)	*Z* = −3.891	<.001
PICU length of stay (days), M (IQR)	14.0 (11.0, 18.0)	11.0 (9.0, 14.0)	*Z* = −3.245	.001
Overall complications, n (%)	31 (41.3)	15 (20.0)	χ^2^ = 7.924	.005
VAP, n (%)	18 (24.0)	7 (9.3)	χ^2^ = 5.928	.015
Pneumothorax, n (%)	6 (8.0)	2 (2.7)	Fisher exact test	.142
CLABSI, n (%)	0 (0)	0 (0)	–	–

CLABSI = central line-associated bloodstream infection, M (IQR) = median (interquartile range), PICU = pediatric intensive care unit, VAP = ventilator-associated pneumonia.

### 3.5. Adverse reactions and safety analysis

**Figure 4.**
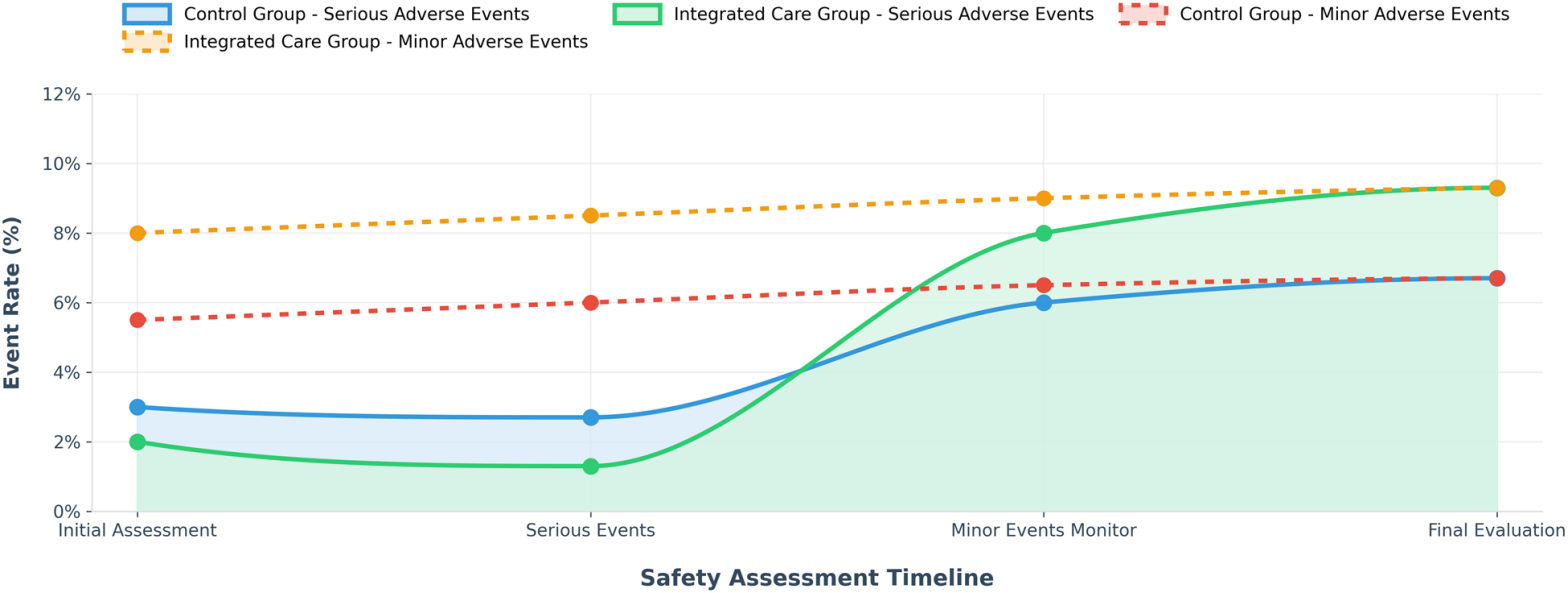
Adverse events and safety profile trends.

The integrated nursing approach demonstrated acceptable safety and tolerability in the current study. There were no significant differences between the 2 groups when it came to unplanned stops in treatment or serious bad events needing quick help, for instance, tubes coming out by accident, serious heart rhythm problems, or significant blood loss. As shown in Table 5 and Figure 4, in the integrated nursing group, 3 cases (4.0%) of mild skin pressure injuries (stage 1) related to nursing care were observed. These skin problems improved after using better positioning techniques and applying special dressings to prevent further damage, without worsening. The other group had 2 cases (2.7%) of similar skin issues, and there was no big statistical difference between the groups (*P* = .500). During the study period, no adverse events occurred that were directly linked to the integrated nursing assessment steps, communication among care teams, or the personalized care plans for patients. Furthermore, the integrated nursing approach did not lead to additional problems, such as trouble breathing, unstable blood flow, or harm to the breathing tube, because of changes made to the nursing plan, including how sedation and pain control were managed and lung-clearing techniques.

**Table 5:** Analysis of adverse events and safety profile.

Safety assessment item	Control group (n = 75)	Integrated care group (n = 75)	Statistical test	*P* value
Serious adverse events (SAEs), n (%)	2 (2.7)	1 (1.3)	Fisher exact test	.5
Unplanned extubation	1	0		
Severe hemodynamic instability*	1	1		
Minor adverse events, n (%)	5 (6.7)	7 (9.3)	χ^2^ = 0.356	.551
Stage 1 pressure injury	2 (2.7)	3 (4.0)		
Transient desaturation (<85%, >1 min)	3 (4.0)	4 (5.3)		
Unplanned treatment discontinuation	0	0	–	–
Events attributable to integrated care protocol	–	0	–	–

* Data presented as number (percentage) of patients. SAEs were defined as events requiring urgent, unplanned medical/surgical intervention. Severe Hemodynamic Instability was defined as requiring escalation of vasoactive support within 1 hour of a care intervention.

## 4. Discussion

This prospective quasi-experimental study evaluated whether an integrated nursing model could improve inflammatory responses, oxygenation, and clinical outcomes in children with severe respiratory failure. We found that, compared withroutine nursing, integrated nursing was associated with a greater decline in IL-6, TNF-α, and CRP over time, faster improvement in oxygenation indices (PaO_2_/FiO_2_ and SpO_2_), shorter mechanical ventilation duration and PICU length of stay, and a lower incidence of complications, particularly VAP. These results indicate that a structured, multidisciplinary nursing pathway may contribute to improved physiologic recovery and clinically meaningful outcomes in critically ill pediatric patients.

The more pronounced reduction in inflammatory markers in the integrated nursing group suggests improved control of systemic inflammation. Prior studies have reported that care bundles and integrated or coordinated nursing approaches may enhance infection prevention, standardize care delivery, and reduce inflammation-related complications in critically ill or respiratory patients.^[11,12]^ In our study, the integrated nursing model emphasized early comprehensive assessment, coordinated care planning, and continuous evaluation, which may facilitate earlier identification and management of factors that aggravate inflammation, such as occult infection progression, inadequate sedation/analgesia, or suboptimal nutrition.^[12–14]^ Importantly, the lower VAP incidence observed in the integrated nursing group is consistent with the concept that standardized respiratory care and prevention bundles can reduce ventilator-associated infections and the downstream inflammatory burden. Together, these findings support that integrated nursing may influence inflammatory trajectories through coordinated risk identification and consistent implementation of preventive measures.

Regarding oxygenation, the integrated nursing group showed a faster and greater improvement in PaO_2_/FiO_2_ from Day 3 onward, along with consistently higher SpO_2_. Existing evidence suggests that protocolized, multidisciplinary respiratory management – including timely airway clearance, appropriate suctioning strategies, positioning, and close ventilator parameter monitoring – can improve gas exchange and reduce ventilator-related harm.^[15–17]^ Our results align with these reports and suggest that the integrated nursing pathway may enhance the timeliness and consistency of respiratory care delivery. In addition, structured collaboration between nurses, physicians, and respiratory therapists may enable faster adjustments to ventilation strategies and earlier optimization of supportive care, which could partly explain the group-by-time differences detected by the mixed-effects models.

Clinically, integrated nursing was associated with shorter mechanical ventilation duration and PICU stay and fewer complications. Similar benefits have been reported in integrated care or multidisciplinary recovery programs in respiratory populations, where coordinated care pathways improve efficiency and support recovery.^[6,18]^ The practical value of our approach is that it operationalizes “integrated care” into a unit-level process, incorporating structured assessment tools, scheduled multidisciplinary discussions, and coordinated implementation and documentation. Such standardization may reduce variability across shifts and staff, strengthen continuity of care, and improve family engagement in pediatric critical care settings.

Several limitations should be considered. First, although data were collected prospectively, group allocation was based on admission period rather than randomization; thus, residual confounding from temporal trends cannot be completely excluded.^[19]^ Second, this was a single-center study with a relatively limited sample size, which may affect generalizability. Third, the intervention was a bundled model, and we could not determine which components contributed most to the observed benefits. Future multicenter randomized or pragmatic trials, together with process evaluations, are warranted to confirm generalizability, evaluate implementation fidelity, and identify the most effective components of integrated nursing pathways in pediatric severe respiratory failure.^[20,21]^

## 5. Conclusion

This study demonstrates that the integrated nursing model, which is based on teamwork across different health areas and focuses on the child patient, is more effective than standard nursing methods for children with severe breathing problems. It helps lower the body’s inflammatory response – evidenced by reduced levels of molecules such as IL-6, TNF-α, and CRP – while also improving lung oxygenation, raising the PaO_2_/FiO_2_ ratio. Integrated nursing was associated with reduced inflammatory marker levels and improved oxygenation in children with severe respiratory failure. It was also associated with shorter mechanical ventilation duration, shorter PICU stay, and fewer complications. These findings support the potential value of an integrated, multidisciplinary nursing model in PICU practice; however, multicenter randomized trials are warranted to confirm causality.

## Acknowledgments

This work was supported by the Hebei Provincial Medical Scientific Research Project Plan (Grant No. 20241933). The authors declare that they have no competing interests.

## Author contributions

**Conceptualization:** Huifen Zhang, Yanhe Ma.

**Data curation:** Yanhe Ma.

**Formal analysis:** Yanhe Ma.

**Investigation:** Yuxiang Wang.

**Methodology:** Lihong Hu, Yuxiang Wang.

**Project administration:** Lihong Hu.

**Software:** Meixian Xu.

**Supervision:** Na Zhang.

**Validation:** Na Zhang, Meixian Xu.

**Writing – original draft:** Huifen Zhang, Yanhe Ma.

**Writing – review & editing:** Yuxiang Wang, Na Zhang, Meixian Xu.
